# Comparative Genome Analysis and Characterization of the Probiotic Properties of Lactic Acid Bacteria Isolated from the Gastrointestinal Tract of Wild Boars in the Czech Republic

**DOI:** 10.1007/s12602-024-10259-7

**Published:** 2024-04-23

**Authors:** Katerina Kavanova, Iveta Kostovova, Monika Moravkova, Tereza Kubasova, Vladimir Babak, Magdalena Crhanova

**Affiliations:** 1 Department of Microbiology and Antimicrobial Resistance, Veterinary Research Institute, Hudcova 296/70, 621 00 Brno, Czech Republic; 2 Department of Experimental Biology, Faculty of Science, Masaryk University, Kamenice 753/5, 625 00 Brno, Czech Republic

**Keywords:** *Limosilactobacillus mucosae*, Probiotics, Wild boars, Pan-genome analysis, pH and bile tolerance, Carbohydrate utilization

## Abstract

Probiotics are crucial components for maintaining a healthy gut microbiota in pigs, especially during the weaning period. Lactic acid bacteria (LAB) derived from the gastrointestinal tract of wild boars can serve as an abundant source of beneficial probiotic strains with suitable properties for use in pig husbandry. In this study, we analyzed and characterized 15 strains of *Limosilactobacillus mucosae* obtained from the gut contents of wild boars to assess their safety and suitability as probiotic candidates. The strains were compared using pan-genomic analysis with 49 *L. mucosae* strains obtained from the NCBI database. All isolated strains demonstrated their safety by showing an absence of transferrable antimicrobial resistance genes and hemolysin activity. Based on the presence of beneficial genes, five candidates with probiotic properties were selected and subjected to phenotypic profiling. These five selected isolates exhibited the ability to survive conditions mimicking passage through the host’s digestive tract, such as low pH and the presence of bile salts. Furthermore, five selected strains demonstrated the presence of corresponding carbohydrate-active enzymes and the ability to utilize various carbohydrate substrates. These strains can enhance the digestibility of oligosaccharide or polysaccharide substrates found in food or feed, specifically resistant starch, α-galactosides, cellobiose, gentiobiose, and arabinoxylans. Based on the results obtained, the *L. mucosae* isolates tested in this study appear to be promising candidates for use as probiotics in pigs.

**Supplementary Information:**

The online version contains supplementary material available at 10.1007/s12602-024-10259-7.

## Introduction

Methods of intensive farming affect the health and well-being of pigs of all ages and often lead to stress and health issues. These factors then contribute to the development of adverse conditions for the establishment of a normal gut microbiota. In pig farming, the post-weaning period is one of the most vulnerable stages in the life of piglets. Animals in this period are highly susceptible to gastrointestinal tract disorders caused by opportunistic pathogens, which typically result in diarrhea, weight loss, and even mortality. These diseases pose significant economic challenges for pig farmers and pork producers worldwide [1].

Post-weaning diarrhea has traditionally been controlled by antibiotic therapy. However, the use of antibiotics and growth promoters for managing post-weaning diarrhea in piglets is currently restricted legislatively in the European Union. Therefore, attention has long been focused on finding suitable alternatives to antibiotics for the control of diarrheal diseases in pigs. Increasingly, new types of probiotics are being used for this purpose [2].

Probiotics, as defined by the Food and Agriculture Organization (FAO) and the World Health Organization (WHO), are live microorganisms that confer health benefits on the host when administered in adequate amounts [3]. Using some suitable probiotics can have a preventive effect against diseases [4] by producing antimicrobial substances that decrease the growth of intestinal pathogens [5] or by stimulating the immune system of animals [2].

Probiotics have to exhibit several properties to survive in the host’s gut environment without causing harm. These properties include the ability to produce organic acids, tolerate bile salts, and adhere to the mucin layer in the intestine among others [2]. They must also meet general safety requirements, such as the absence of transmissible genes for antimicrobial resistance (AMR) and virulence factors [6].

Lactic acid bacteria (LAB), commonly used as probiotics, can have a positive impact on feed utilization in pigs, leading to increased weight gain and improved feed conversion. *Lactobacillus* species are prominent among the bacterial genera frequently employed as probiotics [7].

*Limosilactobacillus mucosae* (formerly known as *Lactobacillus mucosae*) is a gram-positive, heterofermentative LAB. This bacterium has been extensively studied for its probiotic properties, such as its ability to produce exopolysaccharides [8] and the presence of the mucus-binding protein gene (*mub*) [9, 10]. Strains of this species are known for their strong adhesion to mucosal surfaces in the host’s intestine and their capacity to form biofilms in the gut environment [11]. *Limosilactobacillus mucosae* is predominantly found in the gastrointestinal tract (GIT) of pigs [9].

A highly promising source of bacteria with probiotic properties for use in livestock is the digestive tract of wild animals [12–15]. For pigs, the gut of their closest relatives, wild boars, serves as an ideal source of potential probiotic bacteria. Wild boars are not bred under specific artificial conditions like domestic pigs on farms and are not fed commercial diets or subjected to special treatments. It is assumed that the gut microbiota of wild boars is more variable compared to that of domestic pigs, which is likely due to the diet of wild boars, composed primarily of plant components with a lower percentage of carbohydrates and fats, in contrast to the highly nutritious commercial diet of domestic pigs [15]. Furthermore, wild boars are not administered antibiotics or other medications. They may naturally come into contact with antimicrobial components, but their exposure to these substances is significantly lower than that of farm animals [16].

In the current study, 15 strains of *Limosilactobacillus mucosae* isolated from the GIT of wild boars were characterized in terms of safety and beneficial parameters, such as the absence of antimicrobial resistance genes, the ability to survive in gut conditions with typically low pH and the presence of bile salts, the presence of bacteriocins and adhesin-like factors, and variability in the digestibility of carbohydrate substrates. All 15 isolated strains were subjected to comparative genomic analysis together with 49 *L. mucosae* strains retrieved from the NCBI database, and genes of interest were investigated in these isolates. Based on the presence of suitable gene determinants, five candidates of *L. mucosae* were selected for phenotypic in vitro profiling.

## Materials and Methods

### The Origin and Collection of Samples

Samples of colonic content (*n* = 13) and small intestinal content (*n* = 2) were collected from a total of 15 wild boars, each originating from a distinct area of the Czech Republic. These wild boars were legally hunted and shot during regular hunting activities, compliant with Czech Republic legislation, by local hunting associations in the respective areas. Most of the hunting events took place in 2018 and 2019, though five of the strains were sampled in 2013 and 2015. None of the hunted animals displayed any overt clinical signs of disease. All wild boars selected for gut content sampling were adults, consisting of four males and seven females. Information about the sex of four of the wild boars is not known.

### Primary Isolation and Identification of Lactic Acid Bacteria (LAB) Strains

A full bacteriological loop of intestinal content was resuspended in Liquid Man, Rogosa, and Sharp (MRS) culture medium (Oxoid), and the remaining sample was frozen and stored at −70 °C. Cultivation of individual samples was conducted at 37 °C for 48 h under anaerobic conditions in anaerobic jars containing a 10% CO_2_/10% H_2_/80% N_2_ atmosphere facilitated by palladium catalysts (Oxoid, Basingstoke, UK). These cultures were maintained at 37 °C for 48 h using liquid Man, Rogosa, and Sharp (MRS) medium (Oxoid). Subsequently, subculturing was carried out on solid MRS media (Oxoid) under anaerobic conditions, and the subculturing process was repeated until a single morphological type dominated the MRS plate.

Identification of the isolates was accomplished through sequencing analysis of the 16S rRNA gene, employing the primers 16S27f (AGAGTTTGATCMTGGCTCAG) and 16S1492r (TACGGYTACCTTGTTACGACTT) [17].

The PCR products were purified using a QIAquick Purification Kit (Qiagen, Valencia, CA, USA). Subsequently, the PCR amplicons were sequenced in both reverse and forward directions using a Mix2Seq Kit provided by Eurofins Genomics (Luxembourg City, Luxembourg). The resulting sequences were then compared to the GenBank and EzBio Cloud databases (http://www.ezbiocloud.net; accessed on 1 October 2020).

### Whole Genome Sequencing and De Novo Assembly

Whole genome DNA was isolated using a Quick-DNA™ Fecal/Soil Microbe Micropep Kit following the manufacturer’s instructions (Zymo Research, Irvine, CA, USA). The extracted DNA was processed to create a sequencing DNA library using a Nextera Library Preparation Kit (Illumina, Inc, San Diego, CA, USA). Pair-end sequencing was performed on a NextSeq platform utilizing a NextSeq 500/550 High Output Kit v.2.5 (Illumina, Inc, San Diego, CA, USA). To improve data quality, Trim Galore v.0.6.6 (www.bioinformatics.babraham.ac.uk; accessed on 1 December 2020) and Cutadapt v.0.6.6 were employed to trim new read sequences, with Cutadapt v.0.6.6 also used for the removal of low-quality reads. The quality of the read sequences was assessed using MultiQC v.1.9 [18]. De novo genome assembly was accomplished using Unicycler v.0.4.9b [19] and SPAdes v.3.14.1 [20].

### Average Nucleotide Identity

Given the limitations of 16S rRNA sequencing in distinguishing highly related strains, an average nucleotide identity analysis was conducted for all genomic sequences of the isolates. The average nucleotide identity (ANI) calculation was performed using the FastANI tool v.1.32 [21]. As a reference for ANI calculation, the type strain *Limosilactobacillus* *mucosae* DSM 13345 was employed, and its information was accessed from https://lpsn.dsmz.de/. Strains that were not identified as *L. mucosae* according to ANI calculation were compared with 27 type strains of *Limosilactobacillus* spp. (Supplementary Table 1).

### Genome Annotation and Pan-Genome Analysis

Genome annotation was performed for the genomes of the tested isolates originating from the digestive tract of wild boars (*n* = 15), as well as for the genomes of *Limosilactobacillus mucosae* strains obtained from the NCBI database (*n* = 49) (Supplementary Table 2). Gene prediction and annotation were carried out using Prokka v.1.14.6, with searches performed against the following databases: ISfinder, NCBI Bacterial Antimicrobial Resistance Reference Gene Database, and UniprotKB (SwissProt) [22].

Protein sequences annotated by Prokka were utilized for functional annotation through predicted orthology assignments, achieved with the EggNOG-mapper tool e-mapper v.2.1.6-25-g1502c0F [23]. Further comparisons of protein sequences were made using DIAMOND v.2.0.11 protein aligner against EggNoggDB v.5.0.2 [24].

The run_dbcan4 tool (v3.0.4) [25] was employed for the annotation of carbohydrate active enzyme (CAZymes) gene families, with the use of three substrate prediction approaches: HMMER3, DIAMOND, and MMSeq2 [26]. Only genes that were identified by at least two prediction approaches were considered CAZymes genes.

The Prokka-generated files were utilized for pan-genome analysis carried out with ROARY using BLASTP with a percentage sequence identity threshold of 95% [27]. Pan-genome plots were generated using the ROARY create_pan_genome_plots.R script. The determination of an open or closed pan-genome was confirmed using the Micropan R-package [28].

The core genes obtained using the ROARY pipeline were aligned by RAxML-NG to construct a phylogenetic tree based on the general time-reversible model with a model Γ of rate heterogeneity (GTR+G) [29]. The phylogenetic tree generated by RAxML-NG was further adjusted in iTol (https://itol.embl.de). The final plots were edited using R version 4.3.1, and figures were further edited in Inkscape 1.3 software (https://inkscape.org).

### Analysis of Genes Responsible for Defense and Survival Mechanisms

The presence of antibiotic resistance genes in the genomes was assessed using Abricate v.1.0.1 software (https://github.com/tseemann/abricate; accessed on 7 February 2022). The analysis involved a comparison with the following databases: Comprehensive Antibiotic Resistance Database (CARD) [30], ResFinder [31], Argannot [32], Megares [33], and NCBI AMRFinderPlus [34]. Abricate software was configured with a minimum DNA identity threshold of 80% and a minimum sequence coverage of 80%. The BAGEL4 web tool (http://bagel4.molgenrug.nl/) was employed to search for bacteriocin-encoding genes. A combined approach was used for the identification of coding sequences (CDS) encoding adhesins, exopolysaccharide, and bile salt hydrolase (BSH). This involved examining the collective annotation generated by all the annotation tools used (Prokka, e-mapper, run_dbcan4) and local blast searches. The blastn parameters included a minimum identity of 80% and an *E* value of 1e−150. Sequences encoding proteins with adherence ability, such as Lam 29 (from *L. mucosae* ME-340), EF-Tu (*L. reuteri* JCM1081), MapA (*L. reuteri* 104R), MBF (*L. rhamnosus* FSMM22), Mub (*L. reuteri* 1063), and 32-Mmubp (*L. fermentum* BCS87), were explored in all the genomes, including those obtained from NCBI.

### Phenotypic Profiling

The assessment of safety conditions essential for potential probiotics, including antibiotic susceptibility and a blood hemolysis assay, was conducted for all 15 strains isolated from the GIT of wild boars in this study. Based on the previous genomic analysis, and in particular considering the frequency of genes encoding CAZymes in their genomes, five isolates of *Limosilactobacillus mucosae* were selected for additional phenotypic profiling. These selected strains, namely, 6A, 174A, M65A, M212A, and M580A, underwent further testing, which included evaluations of low pH tolerance, bile salt tolerance, and carbohydrate utilization.

### Antibiotic Susceptibility Assay

Antimicrobial susceptibility was assessed using the broth microdilution method in accordance with ISO10932:2010 standards and interpreted following the EFSA FEEDAP Panel Guidance [35]. Microplates inoculated with the tested strains were incubated under anaerobic conditions at 37 °C for 48 h. The minimal inhibitory concentrations (MICs) of specific antimicrobials were determined visually. The antibiotics tested for susceptibility included ampicillin (0.125–16 mg/L), streptomycin (2–256 mg/L), tetracycline (0.5–64 mg/L), erythromycin (0.063–8 mg/L), clindamycin (0.063–8 mg/L), chloramphenicol (0.25–32 mg/L), kanamycin (0.5–2050 mg/L), gentamicin (0.125–512 mg/L), vancomycin (0.25–32 mg/L), and ciprofloxacin (0.125–128 mg/L). *Lactobacillus plantarum* ATCC14917 and *Lactobacillus paracasei* ATC334 strains were included to ensure quality control. The results were assessed and compared against cutoff values established for *Lactobacillus reuteri*.

### Blood Hemolysis Test

The safety of the isolates was assessed by testing their ability to induce the hemolysis of blood cells. Single colonies of the isolates grown on MRS agar plates were inoculated onto Columbia agar plates supplemented with 5% sterile sheep blood (Oxoid, Basingstoke, UK). The plates were then incubated at 37 °C for 72 h in an anaerobic atmosphere. Hemolytic activity on the plates was evaluated visually.

### Low-pH Tolerance Assay

An acid tolerance assay was conducted following an optimized protocol by Ko et al. [36]. The isolates were initially cultivated on solid MRS medium for 48 h at 37 °C under anaerobic conditions. The cultures were subsequently resuspended in a phosphate-buffered saline solution (PBS) with a pH of 7.4 to achieve an optical density at 600 nm equal to 1. Then, 10 ml of the bacterial suspension was inoculated into 40 ml of MRS broth that was acidified to pH 3. Final pH of the inoculatated MRS broth was controlled again. This was followed by incubation at 37 °C under anaerobic conditions for 60, 90, and 120 min. As a control, the same strains were also incubated in MRS broth with a pH of 5.9 and processed in the same manner as the cultures subjected to acidified conditions. After incubation, the bacterial cultures of each strain were diluted, plated on MRS agar plates, and incubated for an additional 48 h at 37 °C under anaerobic conditions, after which the colony-forming units (CFU) were counted. All experiments were performed in triplicate. Survival rate was calculated according to this formula: SR[%] = (CFU/ml after incubation / CFU/ml before incubation) × 100. The final plots of the pH tolerance assay and bile salt tolerance assay were edited using R software (version 4.3.1). The final plots represent means of the measurement in triplicates and the standard deviations of these means.

### Bile Salt Tolerance Assay

The isolates were evaluated for their ability to survive in the presence of various bile salts, including sodium glycocholate, sodium glycodeoxycholate, sodium taurocholate, and sodium taurodeoxycholate (Carl Roth, Germany, and Sigma-Aldrich, USA). The isolates were initially cultivated on solid MRS medium (Oxoid, Basingstoke, UK) for 48 h at 37 °C under anaerobic conditions. Afterwards, they were resuspended in PBS of pH 7.4 to achieve an optical density at 600 nm equal to 1. Ten microliters of each bacterial suspension was added to microplate wells containing 140 µl of MRS broth (Oxoid, Basingstoke, UK) supplemented with bile salts at concentrations of 0, 0.01, 0.1, 0.2, 0.5, 1.0, and 2.0% w/v. The microplates were then incubated for 24 h at 37 °C under anaerobic conditions. After incubation, optical density measurements were taken at a wavelength of 600 nm using a spectrophotometer (Tecan, Männedorf, Switzerland). The growth of these isolates in a medium without bile salts served as a reference, denoted as 100% of growth, and the growth percentage of the isolates in the presence of bile salts was calculated relative to this reference value. All experiments were performed in triplicate. The final plots of the pH tolerance assay and bile salt tolerance assay were edited using R software (version 4.3.1). The final plots represent means of the measurement in triplicates and the standard deviations of these means.

### Analytical Profile Index Test (RAPID ID 32 A)

Analytical profile index (API) tests using RAPID ID 32 A kits (Biomerieux, Marcy-l’Étoile, France) were employed for the phenotype classification of the tested isolates based on their biochemical activity. These tests were conducted following the manufacturer’s instructions.

## Results

### De Novo Genome Assembly and Average Nucleotide Identity

A De Novo Genome Assembly was conducted on the genomes of 15 strains previously identified as *Limosilactobacillus mucosae* based on sequencing of the 16S rRNA gene. The number of assembled contigs varied from 43 to 136, with N50 values ranging between 63,894 and 229,880 bp, and L50 values ranging from 4 to 11 contigs. The assembled genomes of the tested strains exhibited sizes between 1.9 and 2.4 Mbp, with an average GC content of 46.21% (Table 1).

**Table 1:** Assembled genomes and ANI values of *L. mucosae* retrieved from the NCBI database and those isolated in this study

	**Isolate name**	**BioSample**	**Number of contigs**	**Contig size (bp)**	**L50 (contigs)**	**N50 (bp)**	**GC (%)**	ANI (%)*
**Isolates from this study**	6A	SAMN35847944	136	2,213,026	11	63,894	46.10	91.59
	174A	SAMN35847943	82	2,059,905	6	141,186	46.77	97.46
	383A	SAMN35847947	80	2,218,761	6	131,999	46.67	97.31
	598A	SAMN35847945	65	2,051,926	5	154,501	45.94	87.81
	609A	SAMN35847946	71	2,126,623	4	229,880	45.81	88.05
	M65A	SAMN35847952	79	2,154,217	5	194,783	46.56	97.34
	M86A	SAMN35847953	48	2,107,882	4	214,638	45.84	88.02
	M184A	SAMN35847948	84	2,189,144	7	101,904	46.48	97.59
	M193A	SAMN35847949	63	2,091,417	5	176,757	46.86	97.47
	M212A	SAMN35847950	82	2,402,030	7	123,266	45.77	88.55
	M223A	SAMN35847951	43	2,008,450	4	187,471	46.02	88.23
	M387A	SAMN35847939	68	2,135,113	6	129,117	46.71	97.28
	M580A	SAMN35847940	58	1,950,955	5	191,773	45.83	88.04
	M585A	SAMN35847941	49	1,936,352	5	129,350	45.96	88.19
	M592A	SAMN35847942	74	2,258,852	6	177,409	45.83	88.13
**NCBI database**	24WH	SAMN17843086	269	1,747,241	50	7,849	47.30	97.14
	A1	SAMN15515560	4	2,175,821	-	-	46.65	96.60
	AF95-06DT-1A	SAMN31808427	89	2,172,560	7	89,156	46.60	96.18
	AGR63	SAMN02744693	9	1,941,536	3	358,179	47.00	97.15
	AM08-5	SAMN31808526	42	2,207,528	5	202,883	46.60	96.58
	AM96-01DM3TA-r	SAMN31809545	37	2,176,812	5	162,778	46.40	96.23
	BRZ_IG_bin54	SAMEA110134625	21	2,007,845	4	208,415	46.90	97.93
	CRL573	SAMN03081593	38	2,257,701	7	113,254	46.60	96.45
	DPC 6426	SAMN03145820	72	2,079,103	6	91,196	46.70	97.27
	**DSM 13345**	**SAMN02369406**	**91**	**2,254,291**	**9**	**93,868**	**46.40**	**100.00**
	ERR1190914-bin.31	SAMEA7846456	35	2,094,119	7	93,064	46.40	96.67
	F1	SAMN32883541	36	2,035,530	4	170,348	45.90	88.46
	F2	SAMN32883542	25	2,131,655	5	212,822	46.10	89.11
	F4	SAMN32883543	60	2,165,593	7	89,395	46.10	88.39
	F7	SAMN32883544	24	2,117,367	4	239,394	46.70	97.57
	F17	SAMN32883545	48	2,185,230	6	123,438	45.90	88.25
	F20	SAMN32883546	24	2,078,007	4	213,183	46.00	88.50
	F45	SAMN32883547	42	2,259,866	6	120,603	45.80	88.10
	F88	SAMN32883548	31	2,316,090	4	197,825	46.40	97.54
	F108	SAMN32883549	34	2,312,507	4	197,825	46.40	97.49
	F146	SAMN32883550	28	2,263,663	5	148,285	45.60	88.49
	INIA P508	SAMEA5673459	67	2,172,535	9	89,013	46.30	99.47
	KHPC15	SAMN05216545	35	1,880,978	4	167,272	46.70	95.69
	KHPX11	SAMN05216461	39	1,880,857	5	137,576	46.70	95.82
	L1	SAMN14239240	30	1,951,139	6	126,033	46.80	97.20
	L24-B	SAMN10744154	82	2,002,660	5	121,198	46.90	97.45
	LM1	SAMN02470226	2	2,434,610	-	-	46.13	99.93
	MAG15	SAMN15049473	523	1,684,673	118	4,03	47.00	97.95
	Map_17_016	SAMN20301415	116	2,228,339	17	39,176	46.40	96.30
	Map_23_014	SAMN20301627	559	1,829,931	117	4,118	47.00	97.19
	Map_25_015	SAMN20301643	142	2,152,487	16	41,881	46.50	96.50
	Map_27_011	SAMN20301663	63	2,000,573	10	66,075	46.80	96.62
	Map_65_019	SAMN20301977	163	2,165,814	22	29,62	46.60	96.61
	Map_113_023	SAMN20301083	539	1,823,001	121	4,441	47.10	96.93
	Map_122_023	SAMN20301172	71	1,989,192	9	66,066	46.80	96.76
	Map_123_009	SAMN20301178	71	2,024,704	11	68,59	46.80	97.81
	Map_190_006	SAMN20301524	380	2,786,498	18	42,077	45.80	96.32
	MGYG-HGUT-02319	SAMEA5851823	12	2,369,669	3	292,86	46.20	97.20
	OF13-2A	SAMN31809646	42	1,999,487	7	111,192	46.70	96.80
	OF21-12A	SAMN31809666	37	2,240,334	6	173,963	46.40	96.27
	OF23-1pH5A	SAMN31809668	53	2,205,416	5	199,177	46.30	96.58
	OF25-1pH5A	SAMN31809680	41	2,201,492	4	235,664	46.30	96.63
	OF26-1b12A	SAMN31809687	35	1,976,666	5	160,953	46.70	96.57
	OM16-21A	SAMN31809785	82	2,022,804	9	76,478	46.50	96.39
	SUG578	SAMN26525802	26	1,983,623	6	129,254	46.90	97.85
	UN03-219	SAMN31809474	49	1,950,285	6	114,111	46.80	97.35
	UW_TS_LIMLAC1_1	SAMN22412565	60	1,846,668	13	40,201	47.10	97.70
	UW_TS_LIMLAC1_2	SAMN22412566	19	1,684,186	5	121,681	47.20	97.70
	WCC8	SAMN05216430	39	1,880,668	5	137,746	46.70	95.64

ANI values lower than 95% identity with the type strain DSM 13345 are highlighted in underline

Representative genome of *L. mucosae* DSM 13345 is highlighted in bold

ANI comparisons were made between the genome sequences of the isolates tested in this study and the genome of the type strain *Limosilactobacillus mucosae* DSM 13345. The results revealed that the genomes of the isolates 174A, 383A, M65A, M184A, M193A, and M387A exhibited more than 95% identity with the type strain, suggesting that they belong to the same species. However, nine of the isolates tested in this study (6A, M212A, M86A, M592A, 598A, 609A, M580A, M585A, M223A) showed less than 95% identity with the genome of the type strain (Table 1). This suggests a lack of conformity at the species level, even though the sequencing of the 16S rRNA gene identified all tested strains as *Limosilactobacillus mucosae* with identity percentages around 99–100%. ANI calculations were therefore performed for the nine isolates mentioned above, comparing their genomes with other type strain genomes of all available members of the *Limosilactobacillus* genus (*n* = 27) from the NCBI. The ANI values between these strains and type strains did not, however, exceed 95% (Supplementary Table 1).

### Pan-Genome and Phylogenetic Analysis

Genome sequences of 15 *L. mucosae* strains isolated in the current study were used for comparative genome analysis, along with genome sequences of 49 *L. mucosae* strains retrieved from the NCBI database. The ROARY pipeline identified a total of 11,356 distinct orthologous gene groups in the pan-genome (Supplementary Fig. 1). These gene groups were categorized as follows: core genes (312, present in all 64 strains), soft-core genes (374, represented in 61 to 63 strains of the pan-genome), shell genes (2273, found in 10 to 60 strains), and cloud genes (8396, present in 1 to 9 strains).

**Fig. 1:**
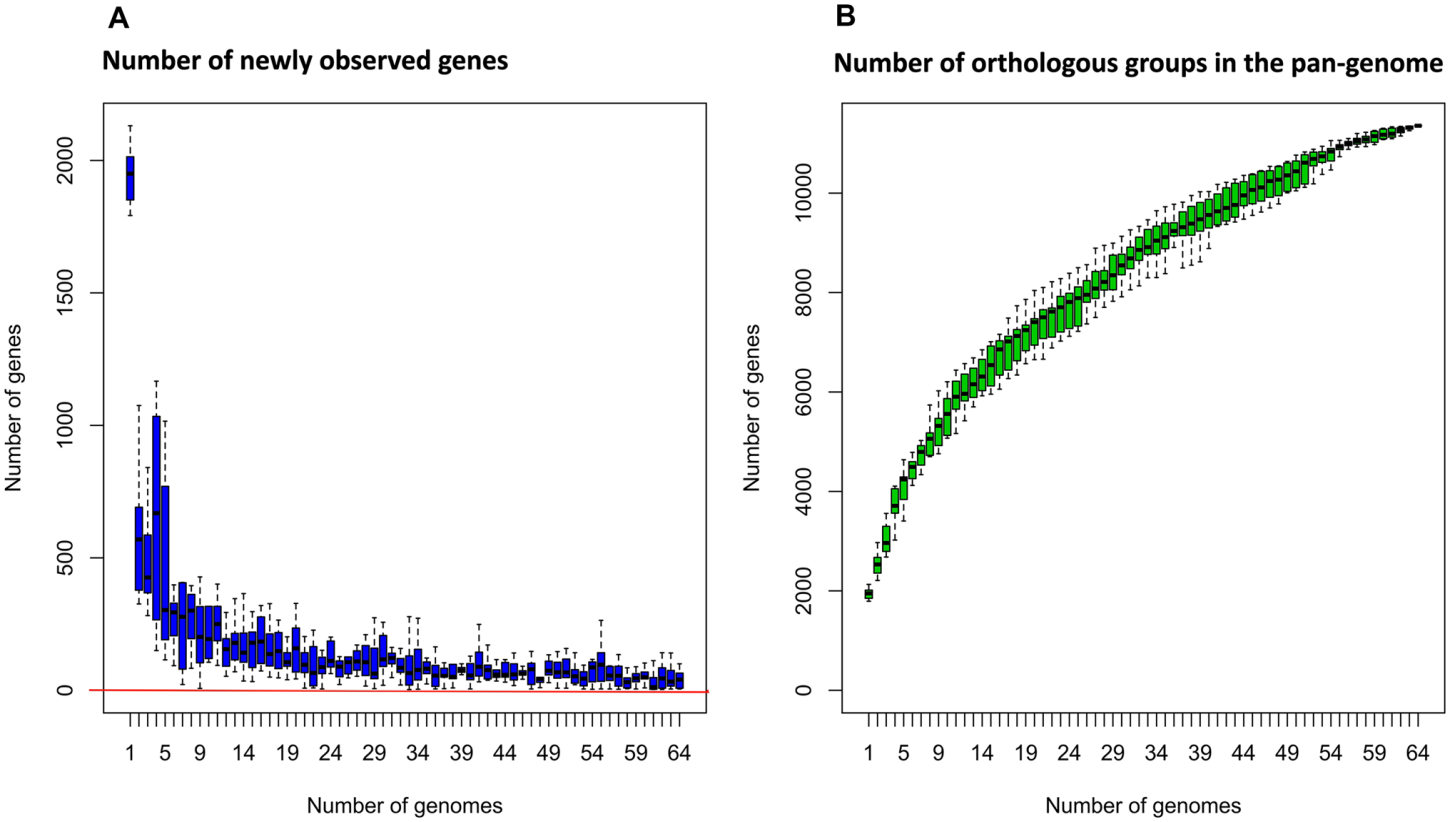
The number of new genes and the number of all orthologous groups in the pan-genome analysis. **A** The number of newly observed genes consistently increases with the addition of each genome to the pan-genome. Notably, the number of new genes remains above zero as more genomes are included. **B** The number of all orthologous groups steadily rises with the inclusion of each additional genome in the pan-genome

The number of newly occurring genes within the pan-genome increased as each additional genome was included in the pan-genome analysis (Fig. 1). This observation suggests that the *Limosilactobacillus* pan-genome can be considered open. To examine this assumption, the data obtained from ROARY were further analyzed using the Micropan R-package. This analysis calculated the alpha value of Heap’s law. The alpha value computed by Micropan was less than 1 (*α* value = 0.99), indicating that the pan-genome of *L. mucosae* is open.

**Fig. 2:**
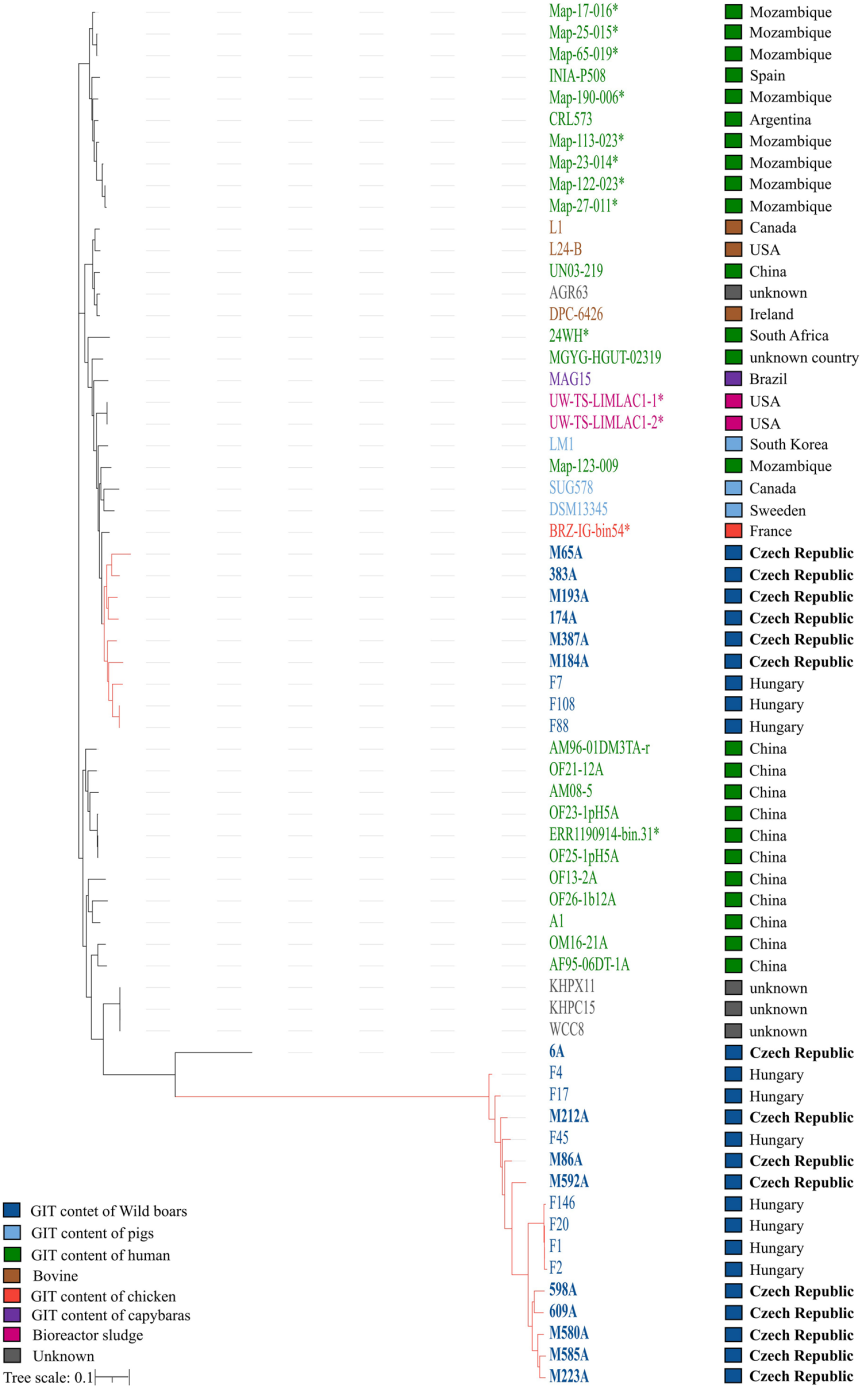
Phylogenetic tree based on core-genome alignment indicating the origin of the *L. mucosae* strains, including their source and geographical location. Branches of the subclusters containing strains from this study are highlighted in red. Strains isolated in this study are highlighted in bold. An asterisk (*) indicates genomes obtained from metagenomic data in public databases

The phylogenetic relationships among individual genomes (*n* = 64) based on core-genome alignment was analyzed by RAxML software (Fig. 2). The first, clearly separated cluster of the phylogenetic tree predominantly includes human GIT content samples from Mozambique, along with one human isolate each from Spain and Argentina. The second cluster is more diverse in terms of origin, containing a subcluster with isolates from bovine GIT, one human isolate from China, and another strain with an unknown origin. Within this cluster, there is an additional subcluster that encompasses the majority of the compared strains and exhibits diversity in origin. This subcluster includes six strains isolated from wild boars, taxonomically identified as *L. mucosae* according to ANI values. It also comprises three isolates from the GIT of wild boars in Hungary, three isolates from the GIT of domestic pigs, and, notably, three strains from human GIT content. Additionally, two strains from bioreactor sludge, one from the GIT of chickens, and one from the GIT of capybaras contribute to the diversity of this subcluster. In contrast, the other two clusters in the phylogenetic tree consist exclusively of human fecal strains from China.

The last, distinctly different cluster in the phylogenetic tree consists of three various subclusters. The first subcluster is formed of two isolates from human GIT, while the second consists of three samples of unknown origin. Interestingly, the third subcluster is formed exclusively of strains from wild boars isolated in this study, exhibiting ANI values lower than 95% with the type strain *L. mucosae* DSM 13345 and seven strains from the GIT of wild boars in Hungary. These strains differ significantly from the rest of the tree, despite the majority of strains being obtained from a similar environment, i.e., the GIT of various organisms.

**Fig. 3:**
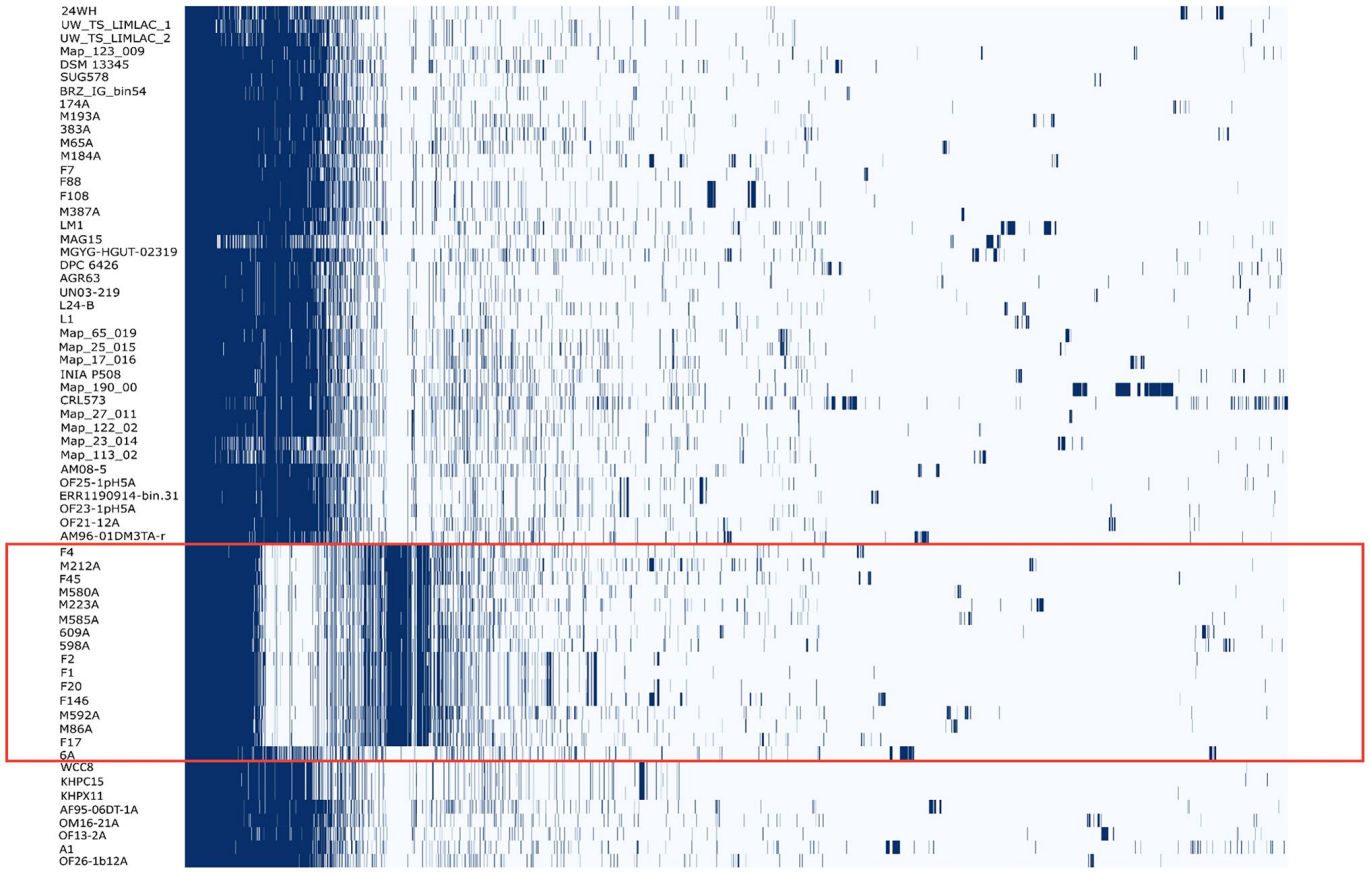
The Roary matrix plot illustrates the presence or absence of genes in each genome of the pan-genome dataset. The strains that were not identified as *Limosilactobacillus mucosae* according to ANI calculation are enclosed in a red frame

It is worth mentioning that the ANI values of isolate 6A not only differ from the *L. mucosae* type strain genome but also from the mentioned subcluster (M212A, M86A, M592A, 598A, 609A, M580A, M585A, M223A, F4, F17, F146, F1, and F2), as depicted in Fig. 3. This strain appears to be more similar to strains with an unknown origin, such as KHPX11, KHPC15, and WCC8, according to the phylogenetic tree.

It is also worth noting that the strains isolated from the gastrointestinal tracts of domestic pigs, namely LM1, SUG578, and DSM 13345, from South Korea, Canada, and Sweden, respectively, exhibit a high degree of similarity to the strains in this study identified as *Lactobacillus mucosae*, which were isolated from the digestive tracts of wild boars in the Czech Republic.

### The Presence of Antimicrobial Resistance Genes

The presence of antimicrobial resistance genes (AMR) in all *L. mucosae* genomes was analyzed using Abricate software. The analysis confirmed the presence of AMR genes only in genomes obtained from the NCBI database. Specifically, the strains L24-B, LM1, Map_17_016, Map_190_006, Map_25_015, Map_65_019, OF13-2A, and OF-26-1b12A showed the presence of AMR genes. Most of these strains originated from the human digestive system, with the exception of strain L24-B, which came from a bovine sample, and strain LM1, which was isolated from the small intestine of a pig.

The AMR genes identified in these strains mainly included the *tet(*W*)* gene (identity = 100.00%), responsible for resistance to tetracycline, the *erm(A)* gene (92.76–98.65% identity), and the *erm(B)* genes (99.87–100.00% identity), which confer resistance to erythromycin. Additionally, the *dfrG* gene (identity = 100.00%), encoding resistance to trimethoprim, and the *lnu(C)* gene (identity = 100.00%), responsible for resistance to lincomycin, were also found.

None of the strains isolated from the digestive tracts of wild boars in the Czech Republic carried any AMR genes in their genomes.

### The Presence of Coding Sequences Encoding Adhesin-Like Factors, Bacteriocins, and Bile Salt Hydrolases

A search was conducted for genes encoding mucin adhesion factors, including Lam29, EF-Tu, MapA, MBF, Mub, and 32-Mmubp, using a local blast pipeline. *lam*29 nucleotide sequences were found in all genomes within the pan-genome dataset, except for strains MAG15 and UW_TS_LIMLAC_2. The coding sequence (CDS) encoding the EF-Tu protein was found in all strains except the MAG15 strain. The CDS for the Mub protein was detected in most strains, with the exceptions being AGR63, ERR1190914-bin.31, F1, F2, F20, F146, OF25-1pH5A, OF26-1b12A, UN03_219, 598A, 609A, and M580A. However, CDS encoding adhesin factors MapA, MBF, and 32-Mmubp were not detected in any of the analyzed genomes.

Furthermore, LPXTG cell wall anchor sequences, along with sortases, anchor cell-surface proteins that operate as adhesins. Genes encoding LPXTG were found in the genomes of 59 strains, with the exceptions being strains 24WH, L1, Map_113_023, and OF-13-2A. Genes for sortase were identified in all genomes of *L. mucosae.*

In addition to these findings, adhesion-like determinants were found within *L. mucosae* genomes. These determinants included genes encoding ComGC protein (present in 29 strains, including four strains from our current study: 6A, 174A, M65A, and M184A), type 4 prepilin-peptidase (found in 46 strains, including 174A, 383A, 6A, M184A, M193A, M387A, and M65A), and fibronectin binding protein in six strains (CRL573, KHPC15, KHPX11, M580A, M585A, and WCC8).

Furthermore, the presence of CDS encoding glycosyltransferases involved in binding exopolysaccharides was also identified. CDS encoding dextransucrase (EC 2.4.1.5, GH70) was found in strains 609A, 6A, M223A, and M65A, and CDS of glycosyltransferase EpsF was detected in strains M223A and M86A.

For all the 64 genomes, a search was conducted for different types of bacteriocins using the BAGEL4 program. Only the CDS encoding enterolysin A was found in 45 strains, including ten isolates from this study, specifically 174A, 383A, M184A, M193A, M212A, M387A, M580A, M592A, M65A, and M86. CDS encoding other types of bacteriocins were not found in any of the analyzed genomes.

CDS encoding bile salt hydrolase (EC 3.5.1.24) was found in all 64 strains of the dataset in one copy.

### Detection of Carbohydrate Active Enzymes (CAZymes)

CAZymes belong to the group of enzymes enabling active metabolism of bacteria by hydrolysis of complex carbohydrates (GHs), their transfer (GTs), or modification (CEs). Annotation revealed GH families belonging to the metabolism of monosaccharides and oligosaccharides such as glucose, galactose, fructose, xylose, or arabinose (GH1, GH2, GH3, GH31, GH32, GH36, GH42, GH43, GH65, GH70). The hydrolysis of polysaccharides such as α-glucans, β-glucans, or arabinoxylans was represented by CAZyme families GH5, GH8, GH13, GH30, GH35, GH39, GH51, GH53, and GH120. Processes connected to cell wall synthesis were represented by CAZyme families GH19, GH23, GH25, and GH73. The most abundant CAZyme families represented in the pan-genome were GH13, responsible for α-glucan hydrolysis, and GH25, participating in cell wall processes.

Carbohydrate binding module (CBM) binds with some carbohydrate hydrolases to enable hydrolysis of polysaccharides. In the dataset, CBM50 alone was identified in all 64 strains, CBM50+GH25 in 16 strains, CBM91+GH43, specifically β-xylosidase, in 14 strains, including isolates 174A and M65A from this study. Further, CBM48+GH13 were identified, specifically amylopullulanase, in all strains except for KHPC15, KHPX11, WCC8, and 1,4-α-glucan branching enzyme in all the dataset except for AGR63, DPC 6426, L1, L24-B, and UN03-219. Finally, CBM34+GH13, specifically neopullulanase, was found in 38 strains, including six isolates from wild boar digestive tract, and intracellular maltogenic amylase in 27 strains, including nine isolates from this study.

Independent enzymes belonging to the GH13 family were also detected: oligo-1,6-glucosidase (EC 3.2.1.10) in 40 strains, including nine strains originating from wild boar intestine, and trehalose-6-phosphate hydrolase (EC 3.2.1.93) in 6A, F4, F17, F45, M212A, M592A, and MGYG-HGUT-02319.

GH2 and GH42 were also a distinct GH family. The GH2 family was represented by β-glucuronidase (EC 3.2.1.31) in 16 tested genomes, including 598A and M86A isolates, while the GH42 family was represented by β-galactosidasae (EC 3.2.1.23), represented in all the pan-genome. The GH36 family, specifically α-galactosidase (mellibiase) enzyme (E.C. 3.2.1.22), was also represented in all 64 strains.

The GH43 family is involved in the cleavage of xylans. Enzymes detected in the pan-genome were xylan-1,4,-β-xylosidase (EC 2.1.1.37) in 34 strains, including six strains from this study, and xylan-1,4,-β-xylosidase with α-arabinodase (EC 3.2.1.37/3.2.1.55) in 16 strains, including M184A, M212A, and M65A isolated from wild boar digestive tract.

**Fig. 4:**
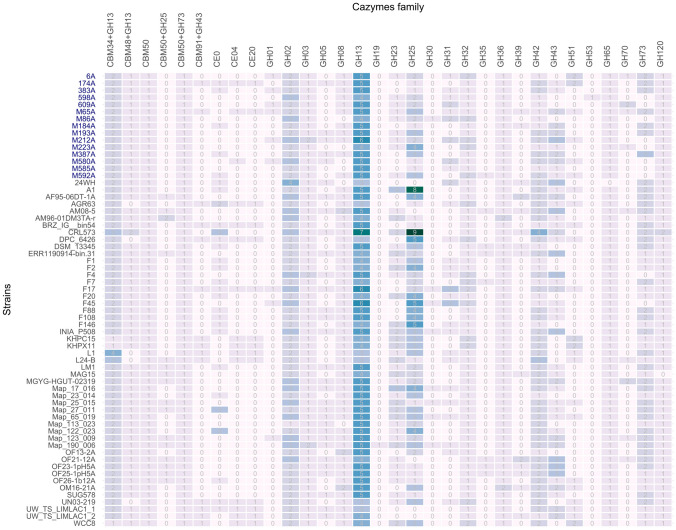
Heatmap of the representation of detected glycoside hydrolase and glycoside esterase families and their copy numbers within the genomes of individual *Limosilactobacillus mucosae* isolates. Strains originating from  the gastrointestinal tract of wild boars in the Czech Republic are highlighted in dark blue

According to the results of CAZyme detection summarized in Fig. 4, the CAZyme distribution among all analyzed genomes of *L. mucosae* is relatively similar with differences in specific strains. The difference is especially visible regarding representation of the GH1 family, which is present only in 6A, 383A, 609A, M65A, M212A, M580A, F17, F45, and Map_123_009. All these strains are isolated from the GIT content of wild boars from the Czech Republic and Hungary, except for Map_123_009, which originated from human GIT content. GH1 is represented by 6-phospho-β-glucosidase, an enzyme that facilitates the hydrolysis of phosphorylated gentiobiose and cellobiose.

## Phenotypic Profiling

### Detection of Safety Requirements in Isolated Strains

The 15 strains of *L. mucosae* were examined for their antibiotic susceptibility profiles and subjected to blood hemolysis assays. Among the tested isolates, 12 strains exhibited MIC values surpassing the cutoff value established for the reference strain *Lactobacillus reuteri* (Table 2). Specifically, strains 6A, 174A, 383A, 598A, M184A, M193A, M223A, M387A, M580A, M585A, M592A, and M65A showed MIC values exceeding the cutoff value for chloramphenicol (8 mg/L). Additionally, strains 598A and M193A exhibited increased MIC values for streptomycin, reaching 128 mg/L. In the blood hemolysis assay, none of the isolates exhibited hemolytic activity after 72 h of incubation under assay conditions.

**Table 2:**
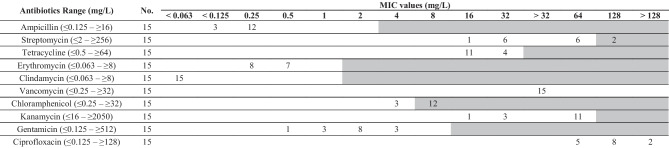
The distribution of MIC values in *L. mucosae* isolates originating from wild boars

The gray zones represent values higher than the MIC cutoff for *Lactobacillus reuteri* [35]

The cutoff values for ciprofloxacin and vancomycin are not known

Representative genome of *L. mucosae* DSM 13345 is highlighted in bold

### Low-pH Tolerance Assay

Three *L. mucosae* isolates (6A, 174A, and M65A) incubated in low pH conditions showed a decrease in bacterial cell counts (CFU/ml) during 2-h incubation. The survival rates of these strains after 120 min of incubation were 34.42%, 7.44%, and 73.84%, respectively. However, the difference in the number of viable cells compared to the control was no more than 1 log unit for any of the three strains. Notably, the survival curve for isolate M65A closely followed the growth curve of the same strain in the control medium.

**Fig. 5:**
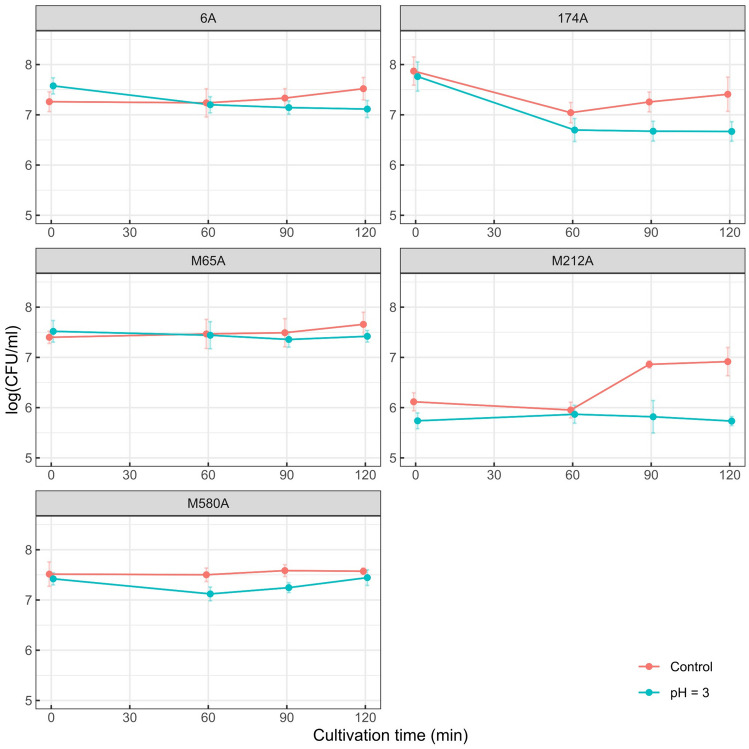
The survival of selected *L. mucosae* strains during incubation at pH 3 and under control conditions at pH 5.9. The data points on the graph represent the geometric means of CFU/ml, and the error bars represent the standard deviations of the means

For strain M212A, although the number of viable cells was approximately 1 log unit lower after 2 h of incubation as compared to the control, the number of CFUs remained stable at pH 3 for 2 h. The survival rate of strain M212A after 120 min of incubation was 100.00%. Similarly, strain M580A exhibited a relatively stable curve of viable cell numbers, with an increase in CFU numbers between the first and second hour of incubation at low pH, approaching those of the control after 2 h of the experiment (Fig. 5). The survival rate of M580A after 120 min of incubation was 98.53%.

### Bile Salt Tolerance Assay

**Fig. 6:**
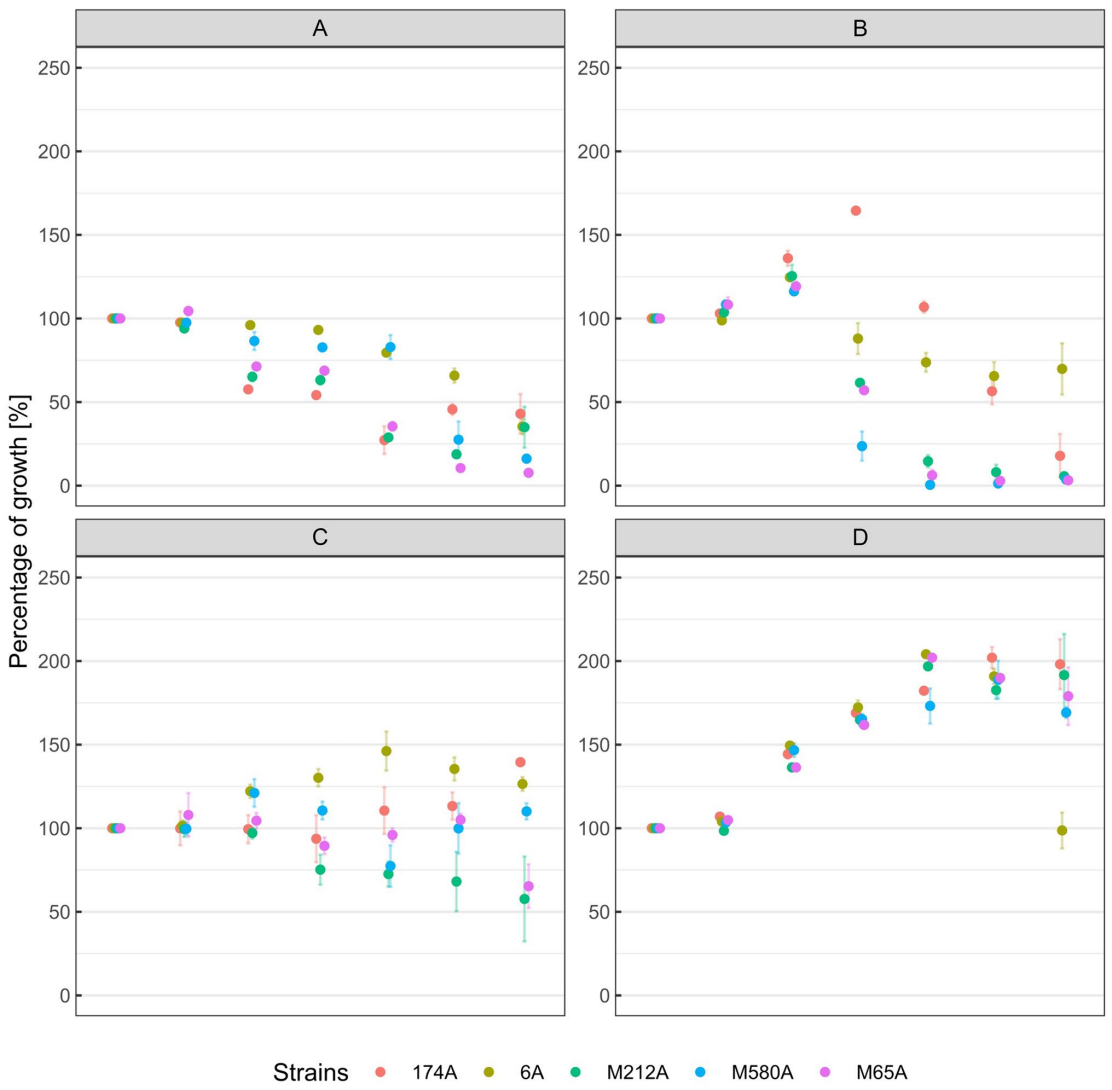
Growth percentage of selected *L. mucoase* isolates in increasing concentrations of bile salts as compared to a control medium. The error bars represent the standard deviations of the means. Sodium glycocholate (**A**), sodium glycodeoxycholate (**B**), sodium taurocholate (**C**), and sodium taurodeoxycholate (**D**)

The ability of five selected isolates to survive in the presence of bile salts was tested at various concentrations of sodium glycocholate, sodium glycodeoxycholate, sodium taurocholate, and sodium taurodeoxycholate (Fig. 6).

The viability of most isolates decreased with increasing concentrations of sodium glycocholate and sodium glycodeoxycholate (ranging from 0.1 to 2.0%). Notably, isolate 174A showed an exception, with its growth percentage increasing to 150% in the presence of 0.2% sodium glycodeoxycholate.

Sodium taurocholate, particularly at concentrations higher than 0.5%, stimulated the growth of isolates 6A and 174A slightly. However, strains M212A and M65A exhibited reduced growth ability in response to increasing concentrations of this salt. All isolates showed growth percentages exceeding 100% in concentrations of sodium taurodeoxycholate higher than 0.1%, except for strain 6A in 2.0% sodium taurodeoxycholate. Importantly, all five strains demonstrated higher viability in taurocholate and taurodeoxycholate salts as compared to glycocholate and glycodeoxycholate salts.

### Carbohydrate Utilization Profiling

**Fig. 7:**
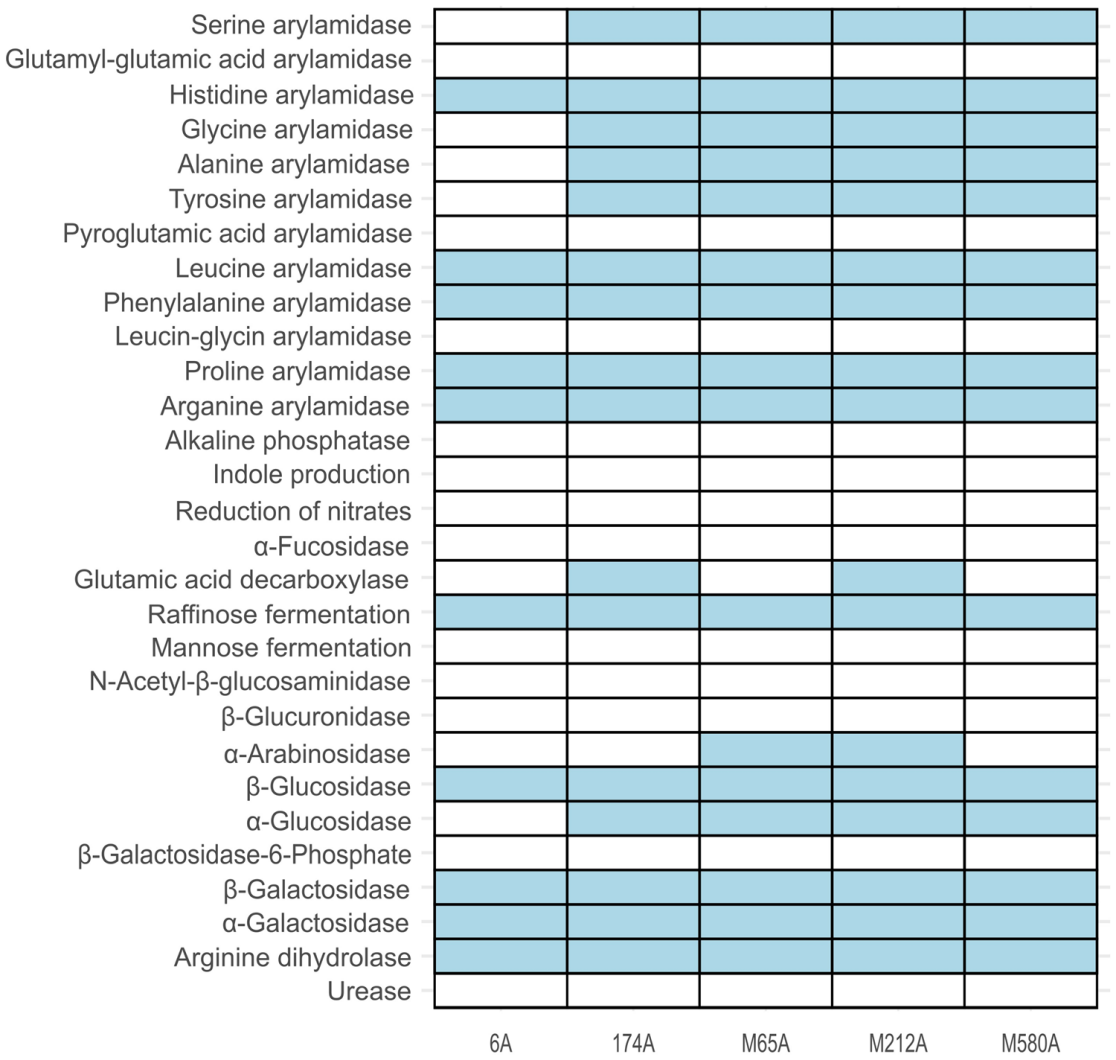
Carbohydrate utilization profiling (RAPID ID 32 A test) of selected *L. mucosae* isolates. The blue color represents a positive reaction; white represents a negative reaction

All five phenotypically tested *L. mucosae* strains exhibited α- and β-galactosidase activity, as shown in Fig. 7. Additionally, all strains demonstrated raffinose fermentation activity, which is consistent with the presence of α-galactosidase.

While all these isolates could degrade β-glucans, α-glucans were degraded by 174A, M65A, M212A, and M580A, but not by strain 6A. Positive utilization of α-arabinans was observed in M212A, M65A, and M580A.

Production of glutamic acid decarboxylase was found in isolates 174A and M212A, with no observed reactions in all isolates for β-galactosidase-6-phosphate, β-glucuronidase, N-acetyl-β-glucosidase, and α-furosidase activity.

## Discussion

The prohibition on using antibiotics as growth promoters and for prophylactic purposes in pig farming is prompting farmers to seek alternatives to replace the effects of antibiotics. One possible alternative is the use of probiotics, for which reason the efforts of researchers have long been focused on the search for and identification of new strains of probiotic bacteria from various suitable sources. For this reason, our study focuses on characterization of the probiotic properties of *Limosilactobacillus mucosae* isolates from the GIT of wild boars in the Czech Republic for potential use in pig husbandry.

Strains of *Limosilactobacillus mucosae* are beneficial lactic acid bacteria known for their probiotic effects on the host’s intestine. It is acknowledged for its health-promoting benefits, including enhanced immunity [37], cholesterol reduction [38], versatile fermentation abilities [8], and its capacity to adhere to mucus [39]. While *L. mucosae* has been identified in various sources, such as the human vaginal tract [8, 40] and milk from sheep [41], goats [42], and donkeys [43], its typical appearance has been described in the GIT contents of humans [44, 45], bovine stool [46], domestic pig GIT contents [9], and the intestinal epithelium of wild boars [14].

The pan-genome analysis of 64 isolates of *L. mucosae* in our study suggests a possible correlation between the similarity of *L. mucosae* strains, their source material, and, potentially, their geographical origin. The pan-genome under examination should be considered an open pan-genome with an *α* value of 0.99. Additionally, the strains in this study that exhibited uncertain taxonomy (strains with an ANI value lower than 95% identity with the type strain) correspond to the open character of the pan-genome. An open pan-genome is characterized by greater variability in phenotype among individual strains, arising from flexible genetic content and the presence of unique genes in each genome [47]. Open pan-genomes are typically associated with bacterial strains that colonize diverse environments, providing more opportunities for the exchange and sharing of genetic material [48]. This aligns with the ability of *L. mucosae* to thrive in complex intestinal environments with various living conditions and to coexist within a diverse microbial community. The open pan-genome of *L. mucosae*, therefore, presents an opportunity to acquire genes that could be advantageous for the utilization of this strain as a probiotic.

When searching for suitable probiotic strains, it is essential to consider not only the appropriate source of isolation and the environmental context from which the isolate originates, but also individual probiotic properties and biosafety. Although none of the 15 isolates from the GIT of wild boars in the Czech Republic showed the production of hemolysins, some strains exhibited MIC values for selected antibiotics higher than the cutoff value of the reference strain. However, genomic analysis revealed no presence of antibiotic resistance genes in any of the strains isolated in this study. The tested strains can, therefore, be considered to be safe and to lack the ability to transfer antibiotic resistance to the intestinal microbiota. Similarly, strains within the pan-genome analysis originating from the GIT of wild boars from Hungary did not contain any antibiotic resistance genes. Conversely, strains of *L. mucosae* primarily sourced from human gut contents, one strain from bovine GIT, and one strain from the GIT of domestic pigs did carry antibiotic resistance genes in their genomes. Bacteria with acquired antibiotic resistance can act as a source of resistance genes for other bacteria in the environment [49]. Resistant lactobacilli originating from domestic pigs, commonly treated with antibiotics such as tetracycline and erythromycin, have already been detected [50–52]. Moravkova et al. conducted a comparison of *L. amylovorus* strains isolated from the GIT of wild boars and domestic pigs, revealing lower levels of resistance to antimicrobial components in wild boars as compared to strains from domestic pigs. Wild boars also did not carry antimicrobial resistance genes *tet(W)* and *erm(B)* in their genomes [53]. The explanation for the observed higher MIC values than the cutoff value, even in strains that do not carry resistance determinants, could be attributed to the fact that the EFSA panel’s cutoff values are not directly established for strains of *L. mucosae*. In this study, strains of *L. mucosae* were assessed based on the cutoff values of *L. reuteri*, which is the most closely related strain in the EFSA panel.

One of the beneficial properties of probiotics is the production of bacteriocins, indicating a competitive advantage of the bacteria in the intestinal environment [54]. In our study, we examined all tested *L. mucosae* genomes, searching specifically for genes encoding bacteriocins. We found that only the gene for enterolysin A, a heat-labile protein belonging to class III bacteriocins [55], was present in the genomes of all isolates. Jia et al. reported the presence of enterolysin A in *L. mucosae* strains from various sources [8]. While our pan-genome study of *L. mucosae* revealed only the presence of enterolysin A, it is important to note that various bacteriocins are known among *Lactobacillus* spp. The bacteriocin helveticin I has been described in *L. helveticus*, lactacin F in *L. johnsonii*, and the plantaricin EF/W/JK/S in *L. plantarum* [56].

The presence of adhesion-like factors was observed through genomic analysis. It was found that CDS encoding lam29 and EF-Tu were present in all strains, except for one for lam29 and two for EF-Tu. This suggests that these proteins are typical for *Limosilactobacillus mucosae*. The presence of lam29 in LAB originating from the gastrointestinal tract of wild boars was confirmed by Keresztény et al. [14]. Several “moonlighting proteins,” including EF-Tu, Lam29, MapA, MBF, Mub, and 32 Mmubp, have also been previously described within the lactobacilli group [57]. While the primary functions of these proteins may differ, they possess a secondary mucin-binding function. The presence of other CDS encoding proteins with adhesive functions, such as ComGC, type 4 prepilin peptidase, and fibronectin-binding protein, was also confirmed in all the compared genomes. The fibronectin-binding protein is crucial for adherence to fibronectin [58]. Proteins like ComGC and type 4 prepilin peptidases are known to be involved in the synthesis of type IV pili, adhesion-like factors typically associated with pathogens [59, 60]. However, their function in lactobacilli is speculative.

In the genomic analysis focusing on the presence of CAZymes, the primary GH families identified were GH13, GH25, and GH2. Microbial enzymes of the family GH13 found in these strains, such as amylopullulanase and oligo-1,6-glucosidase, are responsible for degrading resistant starch, which cannot be decomposed by host enzymes. Specifically, α-amylase hydrolyzes α-1,4-glycosidic linkages, and other α-glycosidases such as pullulanase and oligo-1,6-glucosidase assist in debranching resistant starch [61]. Amylopullulanase exhibits both amylase and pullulanase activities [62]. Amylopullulanase was found in 61 genomes, and oligo-1,6-glucosidase was present in 40 genomes. Phenotypic α-glucosidase activity was observed by API testing on candidate strains in M212A, M580A, M65A, and174A, while it was absent in strain 6A.

Five selected strains phenotypically exhibited α-galactosidase, specifically raffinose, activity and also carried a gene for α-galactosidase in their genomes. The α-galactosides, oligosaccharides mainly found in legumes (such as soybeans), can cause flatulence in the pig and human intestine due to the absence of host α-galactosidases [63]. The digestibility of these less digestible oligosaccharides can then be increased by bacteria of the intestinal microflora capable of producing galactosidase, such as various species of lactobacilli [64].

Phenotypic α-arabinosidase activity was observed in strains M212A and M65A, which was also confirmed by the presence of genes for the enzymes xylan-1,4-β-xylosidase and α-arabinosidase in genomic analysis, indicating that both are capable of degrading arabinoxylans. Arabinoxylans are the primary hemicellulolytic polysaccharides in cereal crops, including corn, wheat, triticale, and oats [65]. While the polymers of arabinoxylans are indigestible for monogastric animals, their oligomers are considered beneficial for gut health and have a positive immunomodulatory effect [66].

Enzyme 6-phospho-β-glucosidase, hydrolyzing the phosphorylated disaccharides cellobiose and gentiobiose, was found in the pan-genome mainly in the strains originated from the GIT of wild boars, including six strains isolated in this study. Cellobiose and gentiobiose are disaccharides that cannot be digested by enzymes in the host’s intestine, but which are degraded primarily by microbial β-glucosidase enzymes [63].

The five phenotypically tested candidate strains can be considered suitable for the digestibility of non-digestible substrates in food or feed, particularly resistant starch, α-galactosides, cellobiose, and gentiobiose. In some cases, they may also be capable of digesting arabinoxylans. The presence of enzymes in selected *L. mucosae* strains responsible for the digestion of less digestible disaccharides, oligosaccharides, and polysaccharides, contributes to their desirable probiotic properties.

The survival of bacteria in stressful conditions when passing through various parts of the host’s digestive tract is one of the most important criteria for the use of probiotic strains [67]. Most of the bacteria colonizing the gut are capable of surviving in hostile environments, such as low gastric pH (with a pH of gastric juice typically ranging from 2 to 3) and the presence of bile salts in the small intestine [68, 69].

When exposed to a simulated test reflecting the transit time through the stomach during food consumption, where they encountered an acidic pH for 120 min, the five selected *L. mucosae* strains demonstrated the ability to survive with a relatively large number of viable cells. Only in strain 174A were the counts of viable cells decreased by more than one log CFU/ml after 120 min of incubation in an acid pH. Nevertheless, the difference in survival ability between a given strain at acidic and control pH was not more than one log during the whole time of measurement. Survival rates of the other four examined strains varied between 34.42% and 100%. The survival of lactobacilli in acidic conditions has been investigated in various studies. For instance, Lee et al. conducted an acid tolerance assay for 1 h at pH 2.5 in *L. reuteri* and *L. gasseri* isolated from human feces, reporting survival rates of 50.2% and 83.5%, respectively [67]. In another study, Kobierecka et al. assessed the viability of *L. salivarius* (30.0–49.7%), *L. plantarum* (30.0–58.5%), and *L. reuteri* (44.7–63.6%), all isolated from the gastrointestinal tract of chickens, at pH 2.5 for 6 h of cultivation [70]. Similarly, Khan et al. tested various lactobacilli from chicken and the human gut, reporting viability ranging from 43 to 99% at pH 2, 3, and 4 after 90 min of incubation [71]. Ting et al. examined *L. salivarius* (survival rate = 83.08%) and *L. agilis* (survival rate = 87.95%) isolated from the gastrointestinal tract of chickens at pH 3 with 2 h of incubation [72]. However, the survival ability of different species within the genus *Lactobacillus* remains unclear, as indicated by the results of these studies. This ambiguity is likely attributed to the diverse methodologies employed in conducting these studies.

When investigating the survival of five selected *L. mucosae* isolates at various concentrations of bile salts, the results indicated that increasing concentrations of glycocholate salts decreased the growth percentage in all tested strains. Conversely, increasing concentrations of sodium taurocholate stimulated the growth of strains 6A and 174A, and sodium taurodeoxycholate had a similar effect on all tested strains. This suggests that the selected *L. mucosae* strains are capable of effectively surviving in the presence of some bile salts, particularly sodium taurodeoxycholate. Similar results were confirmed by Fontana et al. when testing the tolerance of *L. helveticus* in the presence of bile salts. All six tested strains exhibited a similar growth curve in glycocholate, taurocholate, and taurodeoxycholate acids, with a stable growth percentage up to a bile acid concentration of 0.2%. However, the strains showed reduced viability in the presence of glycodeoxycholate [47]. McAuliffe et al. demonstrated the tolerance of *L. acidophilus* to the presence of taurocholate and taurodeoxycholate acids, though the strain was unable to grow in the presence of glycodeoxycholate acid [73]. Similarly, Grill et al. demonstrated the lesser toxicity of taurocholate acids as compared to glycocholate acid in *L. amylovorus* [74].

CDS encoding bile salt hydrolase (EC 3.5.1.24) was found in a single copy of all the examined *L. mucosae* strains. Bile salt hydrolase is one major mechanism of detoxification of bile salts by their deconjugation [75] which leads to a protective effect against toxic conjugated bile acids during intestinal transit. The presence of BSH is considered to be one of the features of probiotics [76].

## Conclusion

In this study, we isolated, identified, and thoroughly characterized *Limosilactobacillus mucosae* strains originating from the digestive tract of wild pigs in the Czech Republic. The open pan-genome of *L. mucosae* suggests its potential for acquiring new genes that enhance survival not only in the environment but also in the host organism, making it a promising probiotic candidate. Our primary focus in this study was, therefore, to evaluate the safety and benefits of *L. mucosae* isolates originating from the GIT of wild boars as probiotics suitable for domestic pigs. In the genomic analysis of all tested strains, we found no transferable antibiotic resistance genes, but identified CDS responsible for bacteriocin production, adhesion, and the breakdown of less digestible oligosaccharides and polysaccharides from feed, which may help to increase feed conversion. Moreover, phenotypic assessments of five selected candidates, based on genotypic analysis, revealed their ability to survive in challenging conditions, including an acidic pH and the presence of bile salts, and also confirmed the findings based on genomic investigation. All these properties make the characterized *Limosilactobacillus mucosae* isolates intriguing candidates for use as probiotics in commercial pig farming.

## Supplementary Information

Below is the link to the electronic supplementary material.

Supplementary file1 (PNG 32 KB)

Supplementary file2 (DOCX 15 KB)

Supplementary file3 (DOCX 18 KB)

## Data Availability

No datasets were generated or analysed during the current study.
