# Supplementary figures and images for: Comparative Genome Analysis and Characterization of the Probiotic Properties of Lactic Acid Bacteria Isolated from the Gastrointestinal Tract of Wild Boars in the Czech Republic
Source: Probiotics Antimicrob Proteins. 2024 Apr 23;17(4):1820–38. doi: 10.1007/s12602-024-10259-7 (PMC12405034; doi:10.1007/s12602-024-10259-7)

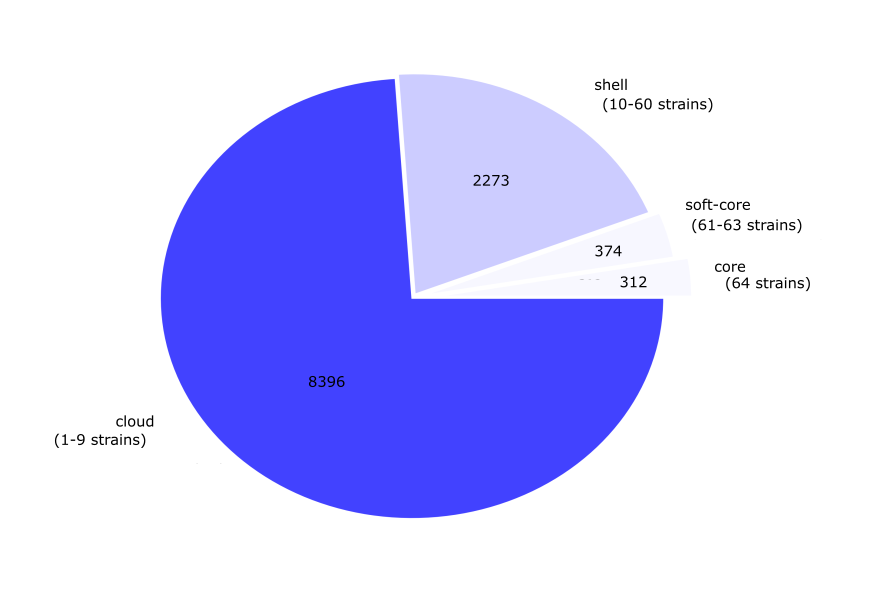

Supplement: Supplementary file 1 — Supplementary file1 (PNG 32 KB) [file 12602_2024_10259_MOESM1_ESM.png]
