# Supplementary material for: Comparative Genome Analysis and Characterization of the Probiotic Properties of Lactic Acid Bacteria Isolated from the Gastrointestinal Tract of Wild Boars in the Czech Republic
Source: Probiotics Antimicrob Proteins. 2024 Apr 23;17(4):1820–38. doi: 10.1007/s12602-024-10259-7 (PMC12405034; doi:10.1007/s12602-024-10259-7)
Supplement: Supplementary file 2 — Supplementary file2 (DOCX 15 KB) [file 12602_2024_10259_MOESM2_ESM.docx]

**Type strains of available *Limosilactobacillus* sp:**

*L. agrestimuris* DSM 106037

*L. agrestis* WF-MT5-A

*L. albertensis* Lr3000

*L. alvi* An810

*L. antri* DSM 16041

*L. avistercoris* Sa3CUN2

*L. balticus* BG-AF-3A

*L. caecicola* DSM 110982

*L. caviae* CCM 8609

*L. coeohominis* DSM 14060

*L. equigenerosi* DSM 18793

*L. fastidiosus* WF-M07-1

*L. fermentum* DSM 20052

*L. frumenti* DSM 13145

*L. gastricus* DSM 16045

*L. gorillae* JCM 19575

*L. ingluviei* DSM 15946

*L. oris* DSM 4864

*L. panis* DSM 6035

*L. pontis* DSM 8475

*L. portuensis* c11Ua_112_M

*L. reuteri* DSM 20016

*L. rudii* STM3_1

*L. secaliphilus* DSM 17896

*L. timonensis* Marseille-P3825

*L. urinaemulieris* c9Ua_26_M

*L. vaginalis* DSM 5837

| **Tested genome** | | **Reference genome** | **ANI value [%]** |
| --- | --- | --- | --- |
| **Isolates from this study** | 6A | *L. portuensis* c11Ua_112_M | 78.20 |
|  | 598A | *L. portuensis* c11Ua_112_M | 79.17 |
|  | 598A | *L. panis* DSM 6035 | 78.27 |
|  | 598A | *L. coleohominis* DSM 14060 | 74.51 |
|  | 609A | *L. portuensis* c11Ua_112_M | 79.21 |
|  | 609A | *L. panis* DSM 6035 | 79.17 |
|  | M86A | *L. portuensis* c11Ua_112_M | 78.39 |
|  | M86A | *L. panis* DSM 6035 | 77.44 |
|  | M212A | *L. panis* DSM 6035 | 78.97 |
|  | M212A | *L. portuensis* c11Ua_112_M | 78.53 |
|  | M223A | *L. portuensis* c11Ua_112_M | 78.59 |
|  | M223A | *L. panis* DSM 6035 | 78.51 |
|  | M223A | *L. pontis* DSM 8475 | 78.51 |
|  | M580A | *L. portuensis* c11Ua_112_M | 78.32 |
|  | M580A | *L. panis* DSM 6035 | 77.33 |
|  | M585A | *L. portuensis* c11Ua_112_M | 79.65 |
|  | M585A | *L. panis* DSM 6035 | 78.62 |
|  | M585A | *L. coleohominis* DSM 14060 | 74.54 |
|  | M592A | *L. portuensis* c11Ua_112_M | 77.79 |
|  | M592A | *L. panis* DSM 6035 | 77.59 |
|  | M592A | *L. coleohominis* DSM 14060 | 74.67 |
| **NCBI database** | F1 | *L. portuensis* c11Ua_112_M | 78.83 |
|  | F1 | *L. coleohominis* DSM 14060 | 74.44 |
|  | F2 | *L. portuensis* c11Ua_112_M | 78.89 |
|  | F2 | *L. panis* DSM 6035 | 77.92 |
|  | F4 | *L. portuensis* c11Ua_112_M | 78.13 |
|  | F4 | *L. panis* DSM 6035 | 77.70 |
|  | F17 | *L. panis* DSM 6035 | 78.59 |
|  | F17 | *L. portuensis* c11Ua_112_M | 78.44 |
|  | F17 | *L. coleohominis* DSM 14060 | 74.57 |
|  | F20 | *L. portuensis* c11Ua_112_M | 78.74 |
|  | F20 | *L. panis* DSM 6035 | 78.27 |
|  | F20 | *L. coleohominis* DSM 14060 | 74.36 |
|  | F45 | *L. portuensis* c11Ua_112_M | 78.81 |
|  | F45 | *L. panis* DSM 6035 | 78.74 |
|  | F45 | *L. coleohominis* DSM 14060 | 74.59 |
|  | F146 | *L. portuensis* c11Ua_112_M | 78.96 |
|  | F146 | *L. panis* DSM 6035 | 78.27 |
|  | F1 | *L. portuensis* c11Ua_112_M | 78.83 |
|  |  |  |  |

**Supplementary Data Table 1:** ANI calculation of strains not identified as L. mucosae compared with 26 type strains of Limosilactobacillus sp. ANI values with threshold less than 70% were not shown. Type strains were obtained from NCBI database.
