# Supplementary material for: Comparative Genome Analysis and Characterization of the Probiotic Properties of Lactic Acid Bacteria Isolated from the Gastrointestinal Tract of Wild Boars in the Czech Republic
Source: Probiotics Antimicrob Proteins. 2024 Apr 23;17(4):1820–38. doi: 10.1007/s12602-024-10259-7 (PMC12405034; doi:10.1007/s12602-024-10259-7)
Supplement: Supplementary file 3 — Supplementary file3 (DOCX 18 KB) [file 12602_2024_10259_MOESM3_ESM.docx]

| **Strain** | **Origin** | **Country** | **BioProject** | **BioSample** |
| --- | --- | --- | --- | --- |
|  |  |  |  |  |
| 24WH | Humuan gut | South Africa | PRJNA684904 | SAMN17843086 |
| A1 | Human gut | China | PRJNA645733 | SAMN15515560 |
| AF95-06DT-1A | Human feces | China | PRJNA903559 | SAMN31808427 |
| AGR63 | - | - | PRJNA239121 | SAMN02744693 |
| AM08-5 | Human feces | China | PRJNA903559 | SAMN31808526 |
| AM96-01DM3TA-r | Human feces | China | PRJNA903559 | SAMN31809545 |
| BRZ_IG_bin54 | Chicken gut | France | PRJEB53581 | SAMEA110134625 |
| CRL573 | Human feces | Argentina | PRJNA262170 | SAMN03081593 |
| DPC 6426 | Bovine | Ireland | PRJNA265168 | SAMN03145820 |
| DSM 13345 | Pig SI | Sweeden | PRJNA222257 | SAMN02369406 |
| ERR1190914-bin.31 | Human gut | China | PRJEB37358 | SAMEA7846456 |
| F1 | GIT of Wild Boar | Hungary | PRJNA926800 | SAMN32883541 |
| F2 | GIT of Wild Boar | Hungary | PRJNA926800 | SAMN32883542 |
| F4 | GIT of Wild Boar | Hungary | PRJNA926800 | SAMN32883543 |
| F7 | GIT of Wild Boar | Hungary | PRJNA926800 | SAMN32883544 |
| F17 | GIT of Wild Boar | Hungary | PRJNA926800 | SAMN32883545 |
| F20 | GIT of Wild Boar | Hungary | PRJNA926800 | SAMN32883546 |
| F45 | GIT of Wild Boar | Hungary | PRJNA926800 | SAMN32883547 |
| F88 | GIT of Wild Boar | Hungary | PRJNA926800 | SAMN32883548 |
| F108 | GIT of Wild Boar | Hungary | PRJNA926800 | SAMN32883549 |
| F146 | GIT of Wild Boar | Hungary | PRJNA926800 | SAMN32883550 |
| INIA P508 | Human feces | Spain | PRJEB32821 | SAMEA5673459 |
| KHPC15 | - | - | PRJEB16859 | SAMN05216545 |
| KHPX11 | - | - | PRJEB17416 | SAMN05216461 |
| L1 | Bovine rectum | Canada | PRJNA609246 | SAMN14239240 |
| L24-B | Bovine | USA | PRJNA515210 | SAMN10744154 |
| LM1 | Pig small intestine | South Korea | PRJNA80653 | SAMN02470226 |
| MAG15 | Capybaras gut | Brazil | PRJNA563062 | SAMN15049473 |
| Map_17_016 | Human gut | Mozambique | PRJNA747761 | SAMN20301415 |
| Map_23_014 | Human gut | Mozambique | PRJNA747761 | SAMN20301627 |
| Map_25_015 | Human gut | Mozambique | PRJNA747761 | SAMN20301643 |
| Map_27_011 | Human gut | Mozambique | PRJNA747761 | SAMN20301663 |
| Map_65_019 | Human gut | Mozambique | PRJNA747761 | SAMN20301977 |
| Map_113_023 | Human gut | Mozambique | PRJNA747761 | SAMN20301083 |
| Map_122_023 | Human gut | Mozambique | PRJNA747761 | SAMN20301172 |
| Map_123_009 | Human gut | Mozambique | PRJNA747761 | SAMN20301178 |
| Map_190_006 | Human gut | Mozambique | PRJNA747761 | SAMN20301524 |
| MGYG-HGUT-02319 | Human gut | - | PRJEB33885 | SAMEA5851823 |
| OF13-2A | Human feces | China | PRJNA903559 | SAMN31809646 |
| OF21-12A | Human feces | China | PRJNA903559 | SAMN31809666 |
| OF23-1pH5A | Human feces | China | PRJNA903559 | SAMN31809668 |
| OF25-1pH5A | Human feces | China | PRJNA903559 | SAMN31809680 |
| OF26-1b12A | Human feces | China | PRJNA903559 | SAMN31809687 |
| OM16-21A | Human feces | China | PRJNA903559 | SAMN31809785 |
| SUG578 | Pig feces | Canada | PRJNA629856 | SAMN26525802 |
| UN03-219 | Human feces | China | PRJNA903559 | SAMN31809474 |
| UW_TS_LIMLAC1_1 | Bioreactor sludge | USA | PRJNA768566 | SAMN22412565 |
| UW_TS_LIMLAC1_2 | Bioreactor sludge | USA | PRJNA768569 | SAMN22412566 |
| WCC8 | - | - | PRJEB16233 | SAMN05216430 |
|  |  |  |  |  |

* NZ_AHIT01000000 NZ_AHIT01000001-NZ_AHIT01000055

| **Strain** | **Origin** | **Country** | **BioProject** | **BioSample** |
| --- | --- | --- | --- | --- |
|  |  |  |  |  |
| 6A | Intestinal contents of wild boar | Czech Republic | PRJNA886611 | SAMN35847944 |
| 174A | Intestinal contents of wild boar | Czech Republic | PRJNA886611 | SAMN35847943 |
| 383A | Intestinal contents of wild boar | Czech Republic | PRJNA886611 | SAMN35847947 |
| 598A | Intestinal contents of wild boar | Czech Republic | PRJNA886611 | SAMN35847945 |
| 609A | Intestinal contents of wild boar | Czech Republic | PRJNA886611 | SAMN35847946 |
| M65A | Intestinal contents of wild boar | Czech Republic | PRJNA886611 | SAMN35847952 |
| M86A | Intestinal contents of wild boar | Czech Republic | PRJNA886611 | SAMN35847953 |
| M184A | Intestinal contents of wild boar | Czech Republic | PRJNA886611 | SAMN35847948 |
| M193A | Intestinal contents of wild boar | Czech Republic | PRJNA886611 | SAMN35847949 |
| M212A | Intestinal contents of wild boar | Czech Republic | PRJNA886611 | SAMN35847950 |
| M223A | Intestinal contents of wild boar | Czech Republic | PRJNA886611 | SAMN35847951 |
| M387A | Intestinal contents of wild boar | Czech Republic | PRJNA886611 | SAMN35847939 |
| M580A | Intestinal contents of wild boar | Czech Republic | PRJNA886611 | SAMN35847940 |
| M585A | Intestinal contents of wild boar | Czech Republic | PRJNA886611 | SAMN35847941 |
| M592A | Intestinal contents of wild boar | Czech Republic | PRJNA886611 | SAMN35847942 |
|  |  |  |  |  |

**Supplementary Data Table 2:** Origins of L. mucosae obtained from NCBI database and from wild boars
